# Culture systems influence the physiological performance of the soft coral *Sarcophyton glaucum*

**DOI:** 10.1038/s41598-020-77071-5

**Published:** 2020-11-19

**Authors:** Tai-Chi Chang, Anderson B. Mayfield, Tung-Yung Fan

**Affiliations:** 1 Institute of Marine Biology, National Dong Hwa University, Pingtung, 944 Taiwan; 2 National Museum of Marine Biology and Aquarium, Pingtung, 944 Taiwan; 3 Atlantic Oceanographic and Meteorological Laboratory, National Oceanic and Atmospheric Administration, Miami, FL 33149 USA; 4 Cooperative Institute for Marine and Atmospheric Studies, University of Miami, Miami, FL 33149 USA

**Keywords:** Physiology, Zoology

## Abstract

There is an urgent need to develop means of ex situ biobanking and biopreserving corals and other marine organisms whose habitats have been compromised by climate change and other anthropogenic stressors. To optimize laboratory growth of soft corals in a way that could also benefit industry (e.g., aquarium trade), three culture systems were tested herein with *Sarcophyton glaucum*: (1) a recirculating aquaculture system (RAS) without exogenous biological input (RAS−B), (2) a RAS with “live” rocks and an exogenous food supply (RAS+B), and (3) a simple flow-through system (FTS) featuring partially filtered natural seawater. In each system, the effects of two levels of photosynthetically active radiation (100 or 200 μmol quanta m^−2^ s^−1^) and flow velocity (5 or 15 cm s^−1^) were assessed, and a number of soft coral response variables were measured. All cultured corals survived the multi-month incubation, yet those of the RAS−B grew slowly and even paled; however, once they were fed (RAS−B modified to RAS+B), their pigmentation increased, and their oral discs readily expanded. Light had a more pronounced effect in the RAS−B system, while flow affected certain coral response variables in the FTS tanks; there were few effects of light or flow in the RAS+B system, potentially highlighting the importance of heterotrophy. Unlike the ceramic pedestals of the FTS, those of the RAS+B did not regularly become biofouled by algae. In concert with the aforementioned physiological findings, we therefore recommend RAS+B systems as a superior means of biopreservating and biobanking soft corals.

## Introduction

Certain soft corals and all reef-building corals form associations (collectively termed “holobionts”) with symbiotic dinoflagellates and bacteria; these microbes are critical to the physiological function and survival of their animal hosts^1−3^. Although the mutualistic dinoflagellates translocate photosynthetically fixed carbon into host cytoplasm, corals and other endosymbiotic anthozoans nevertheless rely on heterotrophy, as well, for nourishment^4−5^. These mutualistic associations consequently span a bridge between benthic and planktonic food webs and consequently play critical roles as primary and secondary producers^6^.

The culture of alcyonacean soft corals (Cnidaria: Anthozoa: Octocorallia: Alcyonacea)- notably *Sarcophyton*, *Sinularia*, and *Lobophyton*- has advanced rapidly in recent decades given the needs to (1) explore the impacts of environmental change on their physiological performance and (2) develop a sustainable supply for both the marine cosmeceutical and aquarium trade industries^1,7,8^. Many cultured soft corals used in natural product research rely on natural seawater flow-through systems (FTS)^9,10^; however, natural product yields may differ from those of wild populations^11^. Other studies have used recirculating aquaculture systems (RAS) in which seawater quality parameters can be readily and automatically modulated by a variety of microprocessor-based dosing systems^12−14^. While biological factors, such as “live rocks” (typically dead coral skeleton encrusted by crustose coralline algae) that accumulate rich microbial flora^15,16^ and heterotrophic feeding^4−5,17^, have been explored more recently, a systemic comparison across different types of culture systems has not yet been achieved for soft corals.

Light intensity and flow velocity have been examined in numerous soft coral experiments^7,17−19^. The former is especially important for soft corals harboring dinoflagellates, whose photosynthetic performance can directly or indirectly affect the physiology and growth of their hosts^20,21^. Light has also been shown to affect not only growth, but also secondary metabolite production, in symbiotic soft corals^19^. With respect to flow, soft corals alter their morphologies in response to changes in seawater velocity^18^, and seawater flow promotes material exchange and metabolism (thereby affecting metabolite yield). However, only single-factor experiments have been conducted with soft corals, despite the well-documented interaction between irradiance and water flow in hard corals like *Galaxea fascicularis*^22^. Thus, research focused on the interaction of light and flow on soft coral physiology is needed.

Measurements of morphology and growth in soft corals are more difficult compared to stony corals^23^; the latter consist of a large amount of calcium carbonate (exoskeleton) covered by a thin veneer of live tissue. Alcyonacean soft corals, in contrast, are mostly fleshy, with only small amounts of calcium carbonate (sclerite) skeleton. Shape is instead maintained by hydrostatic pressure from water pumped into a canal system. Such hydroskeletons change quickly in size and shape in response to environmental shifts. Therefore, it is difficult to accurately measure their size and growth^23^, though oral disc diameter (ODD), stalk diameter, colony height, and wet and dry colony weight have all been assessed in prior works^24,25^. In general, ash weight and ash-free dry weight (AFDW) are thought to represent skeleton and organic matter content, respectively^25^.

Soft corals of the genus *Sarcophyton* are abundant on many coral reefs in the Indo-Pacific Ocean, and particularly Taiwan^6^. *Sarcophyton* colonies are mushroom-shaped, with (1) fleshy stalks elevating the polyp-bearing oral discs and (2) sclerites in the interior fleshy tissues of the colony. *Sarcophyton glaucum* (Quoy & Gaimard, 1833) is a suspension feeder that lives in mutualistic association with phototrophic dinoflagellates (family Symbiodiniaceae), as well as an array of other microbes. It has been cultured in FTS to investigate natural product production^9^ and in RAS to assess the effects of light intensity and flow velocity on its physiology^17,20,26,27^. Given the recent recommendation to develop the capacity to biobank and biopreserve corals ex situ^28^, a rigorous comparison of soft coral performance across different culture systems is of need.

**Figure 1:**
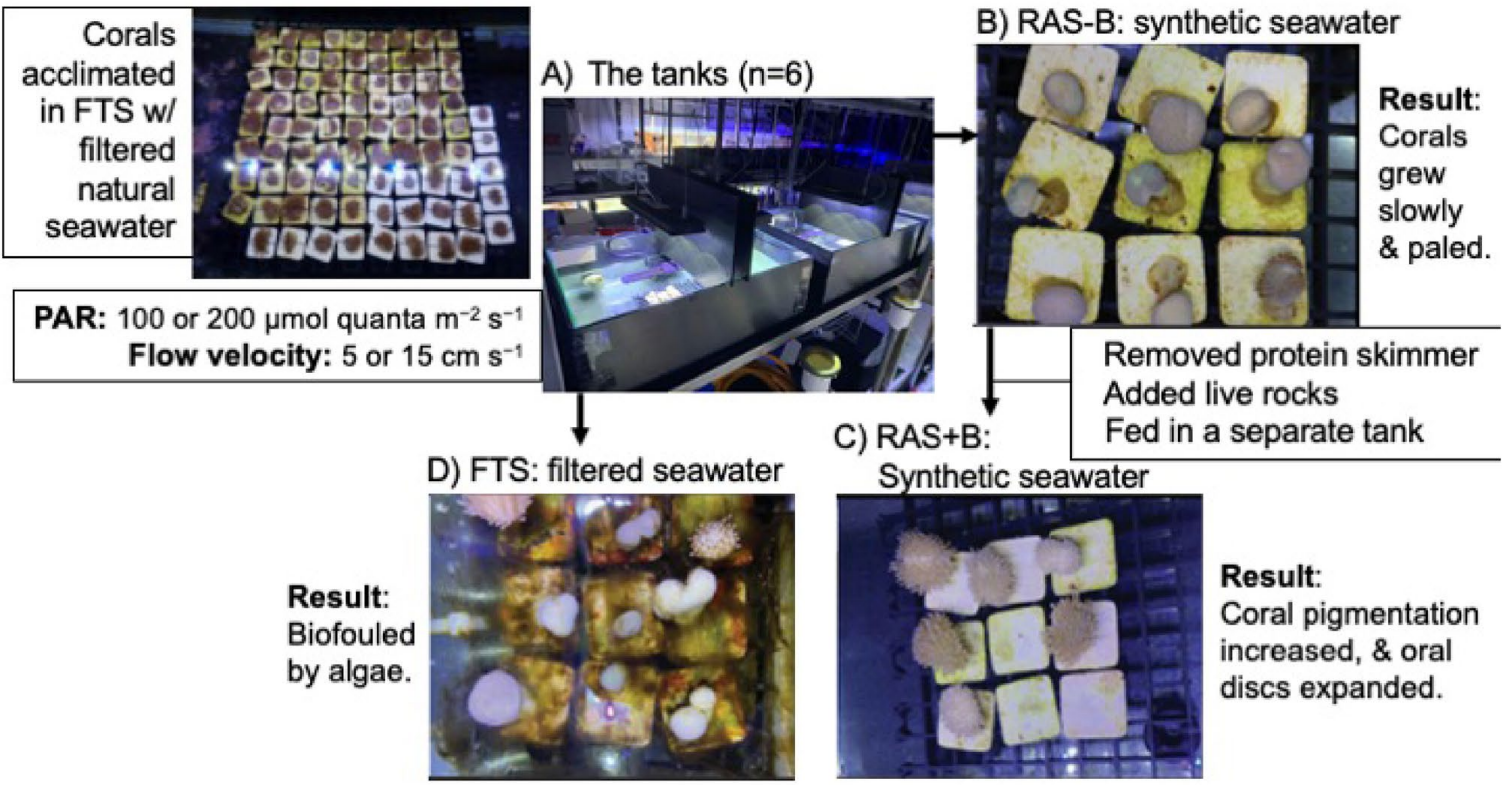
Schematic of experimental design alongside key findings. An image of soft corals mounted to ceramic pedestals and the tank system (**A**). Representative pedestals and soft coral fragments have been shown from the RAS−B (**B**), RAS+B (**C**), and FTS (**D**).

Herein we aimed to study the effect of three different types of culture systems—a RAS with no microbial flora or exogenously supplied food [RAS minus (−) additional biological input = RAS−B], a RAS without a protein skimmer supplemented with live rocks and a supplemental phytoplankton solution fed corals [RAS plus ( +) additional biological input = RAS+B], and a traditional FTS- on the physiological performance of *S. glaucum* (Fig. 1). Within each culture system, two levels of photosynthetically active radiation (PAR; 100 or 200 μmol quanta m^−2^ s^−1^) and flow velocity (5 or 15 cm s^−1^) were administered, and several coral physiological response variables were measured to assess performance: colony color, height, base diameter, growth rate, ODD, and percent (%) organic weight (i.e., AFDW). We hypothesized that the physiological performance of the corals would be superior in the RAS+B system due to the (1) potential for heterotrophy and (2) the fact that feeding was carried out in separate feeding tanks (which would potentially limit macroalgal growth in the culture tanks).

## Results

### Water quality

The pH, concentrations of Ca^2+^and Mg^2+^, and carbonate hardness/alkalinity (KH) were significantly higher in RAS−B and RAS+B than in FTS (Table 1). Levels of ammonia, nitrite, nitrate, and phosphate in the three systems remained below detectable levels (< 0.2 mg L^−1^) during the entire experimental period (data not shown). PAR and flow did not deviate significantly over time (*p* > 0.10) and remained close to their target values (see details in “Methods”).

**Table 1:** Comparison of seawater chemistry parameters across the three culture systems: RAS−B (*n* = 13 measurements), RAS+B (*n* = 9), and FTS (*n* = 9).

Culture system	Temperature (°C )	Salinity	pH	Ca^2+^ (mg L^−1^)	Mg^2+^ (mg L^−1^)	KH (dKH)
RAS−B	25.7 ± 0.10^B^	34.8 ± 0.10	8.15 ± 0.03^A^	450 ± 4.80^A^	1318 ± 6.32^AB^	7.4 ± 0.15^A^
RAS+B	26.0 ± 0.11^B^	34.9 ± 0.10	8.11 ± 0.03^AB^	447 ± 5.27^A^	1320 ± 5.00^A^	7.3 ± 0.17^A^
FTS	26.5 ± 0.08^A^	35.0 ± 0.10	8.03 ± 0.02^B^	411 ± 4.55^B^	1297 ± 4.41^B^	7.0 ± 0.13^B^

When the non-parametric ANOVAs detected differences across the culture systems, Dunn’s multiple comparisons tests were carried out between individual means, and significant differences (*p* < 0.05) are denoted by capital letters. All error terms represent standard error of the mean.

### Coral health and growth

**Figure 2:**
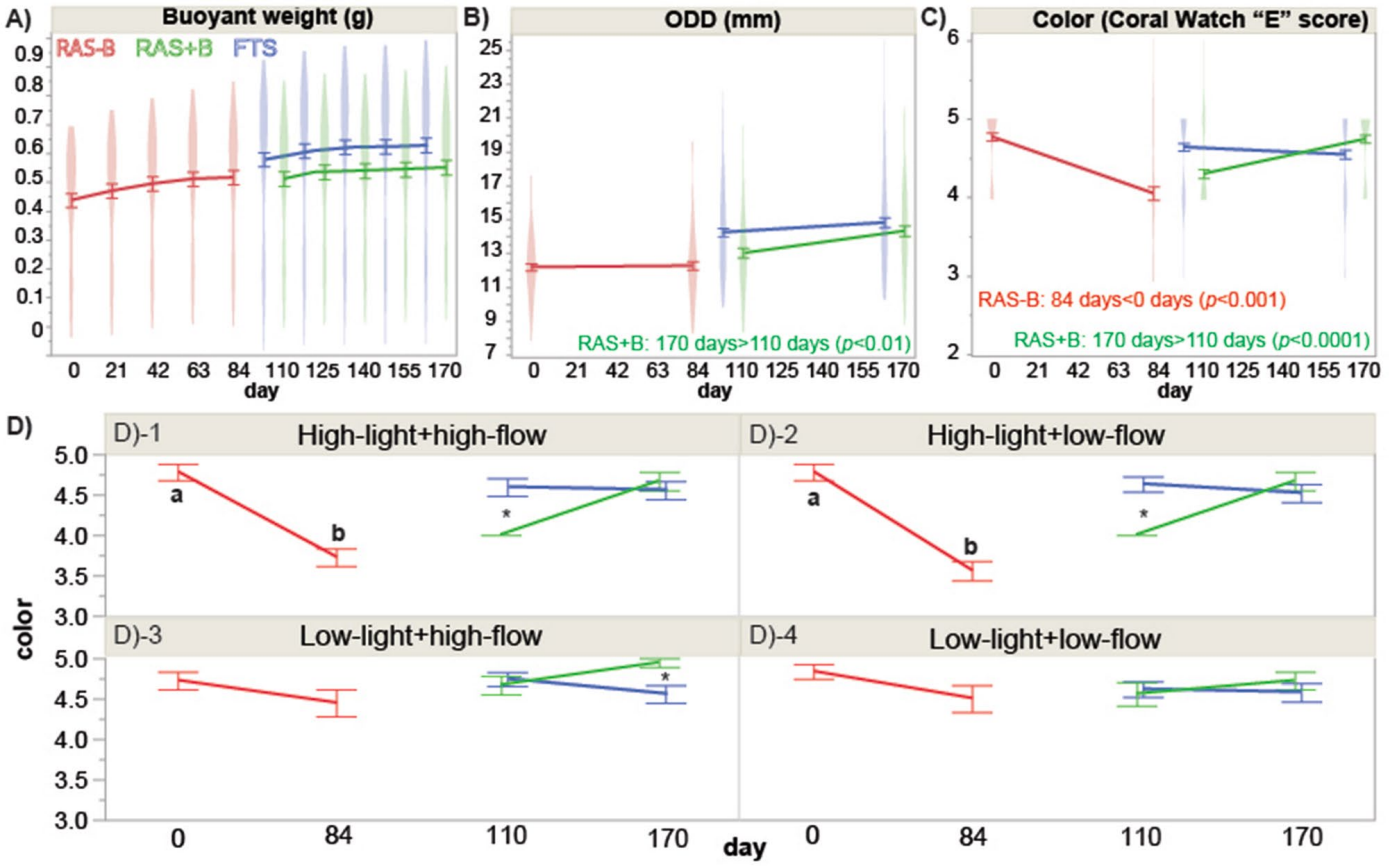
Violin plots depicting temporal variation in buoyant weight (**A**), oral disc diameter (ODD: **B**), and fragment color (**C**) of soft corals over time in the three culture systems—recirculating aquarium system (RAS) without additional biological input (RAS−B; red), RAS with exogenous food supply and “live” rocks (RAS+B; green), and a flow-through system (FTS) featuring partially filtered seawater (blue)—as well as interaction plots of raw color data (**D**). It should be re-emphasized that RAS−B tanks were converted to RAS+B ones after day-84. Error bars spanning mean values at each sampling time represent standard error, asterisks in (**D**) denote Tukey’s honestly significant differences (*p* < 0.05) between RAS+B and FTS corals, and lowercase letters in (**D**) denote temporal differences (Tukey’s HSD, p < 0.05) in color for corals of the RAS−B culture system. RAS+B vs. FTS differences in (**D**) are instead denoted by asterisks (*).

Although all fragments in the three culture systems were mushroom-shaped (Fig. 1B–D), many soft coral fragments cultured in the RAS−B system appeared unhealthy (Fig. 1B); polyps were not always extended, and neither their buoyant weight [BW; Fig. 2A; one-way ANOVA effect of time (for this and all following tests in this paragraph), *F* = 1.77, *p* = 0.134] nor their ODD (Fig. 2B; *F* = 0.0629, *p* = 0.8023) increased significantly over time. They also paled significantly over this period when pooling across all light × flow treatments (Fig. 2C; *F* = 53.1, *p* < 0.0001), though the color decrease of RAS−B corals of the two low-light treatments [high-light + low flow (Fig. 2D-1) and high-light + high flow (Fig. 2D-2)] was not statistically significant. After changing to the RAS+B system (see Fig. 1C for representative fragment images.), their ODD enlarged (Fig. 2B; *F* = 9.54, *p* < 0.01), and their pigmentation increased (Fig. 2C; *F* = 32.8, *p* < 0.0001). Green, filamentous algae tended to overgrow the pedestals in the FTS (Fig. 1D), and neither buoyant weight (Fig. 2A; *p* = 0.635) nor ODD (Fig. 2B; *p* = 0.120) increased significantly between culture days 110 and 170. FTS fragment color actually decreased over this same period (Fig. 2C), though the global temporal difference was not statistically significant (*p* = 0.204; see also Fig. 2D for temporal changes for each of the four light × flow interaction groups.).

**Figure 3:**
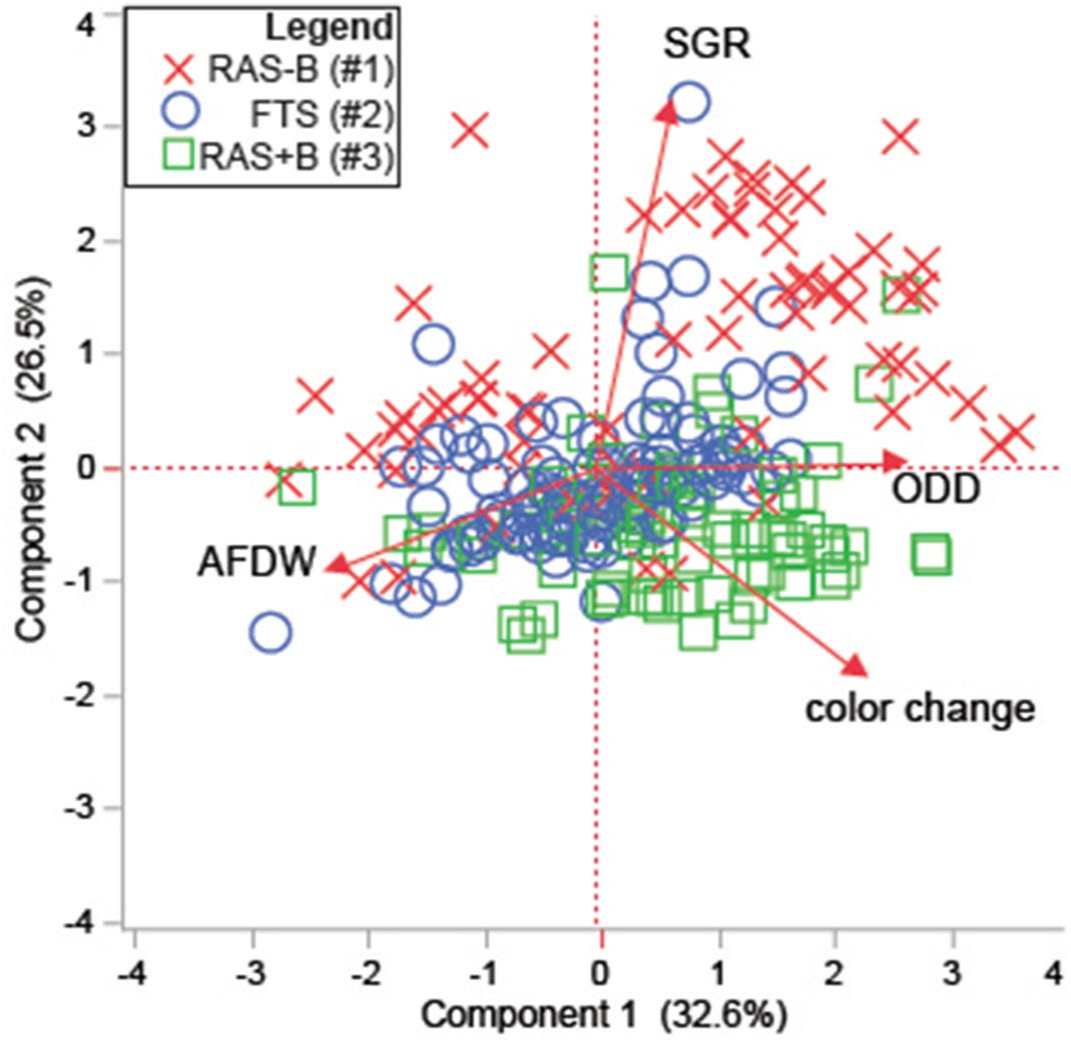
Principal components analysis of correlations across standardized soft coral physiological response variable data. Please note that specific growth rate (SGR), color change, and oral disc diameter (ODD) all reflect rates or changes over time (day^−1^, final–initial, and % change day^−1^, respectively); final values were instead incorporated for percent (%) organic weight (i.e., AFDW), which was only measured once. There was a clear inverse relationship between % ODD change day^-1^ and AFDW.

In the principal components analysis (PCA) on correlations (Fig. 3), the first two axes captured nearly 60% of the variation in the dataset, and the primary coordinate (x) axis featured ODD change (eigenvalue = 0.63) and color change (0.55) as the dominant positive loading factors, with AFDW as the dominant negative one (− 0.53). The secondary (y) axis was primarily defined by the negative relationship between the SGR (0.44) and the color change (− 0.48). There was evident separation between RAS−B and RAS+B samples in the PCA, and a multivariate ANOVA (MANOVA) + canonical correlation analysis (CCA) verified this (Wilks’ lambda of culture treatment effect = 16.6, *p* < 0.001). A more detailed explanation on how the individual treatments (light × flow) affected these soft coral response variables can be found in Fig. 2D, Table 2 (statistically significant findings only), the online supplemental results (OSR), Supplementary Table S1 (the full repeated-measures ANOVA model), and Supplementary Figs. S1 and S2. Briefly, there were more significant effects of light on response variables measured in corals of the RAS−B [e.g., ODD growth (Supplementary Fig. S2G) and color change (Fig. 2D, Supplementary Fig. S2M)]. Flow more significantly affected the response of soft corals cultured in the FTS, particularly the AFDW (Supplementary Fig. S2L), which was higher at low flow rates. In contrast, neither light nor flow affected the SGR (Supplementary Fig. S2E) or ODD growth (Supplementary Fig. S2H) of RAS+B corals. As an exception, the color increase was higher for RAS+B corals cultured at the higher light level (Supplementary Fig. S2N), though no significant post-hoc differences were noted.

**Table 2:** Select two-way, repeated measures ANOVA results for the effects of culture system, light, flow, and their interaction(s) on several soft coral response variables.

Response variable effect	df	Exact *F*	*p*
**Buoyant weight (increase day**^**−1**^**)**^**a**^			
Culture system (see Fig. 2A for raw data.)	2	49.1	< 0.0001
Culture system × light	2	6.30	0.00330
Culture system × flow	2	4.36	0.0173
**Colony height (cm; final value only) (Supplementary Fig.** S1**A–D,** S2**B,C)**			
Flow	1	63.01	< 0.0001
Culture system × flow	1	7.60	0.0065
Culture system × light × flow	1	7.44	0.0071
**SGR (day**^**−1**^**)**^**b**^** (Supplementary Figs.** S1**E–H,** S2**D–F)**			
Culture system	2	55.6	< 0.0001
Culture system × flow	2	5.33	0.0075
Light × flow	1	10.20	0.0023
**ODD (% change day**^**−1**^**) [Supplementary Figs. **S1**I–L**, S2**G–I, and Fig. ****2****B (raw data)]**			
Culture system	2	7.12	0.0017
Culture system × light	2	3.61	0.0334
**Percent organic weight (AFDW)**^**b**^ **(Supplementary Figs. **S1**M–P,** S2**J–L)**			
Culture system	2	14.2	< 0.0001
**Color change (final-initial) (Supplementary Figs.** S1**Q–T,** S2**M–O**^**c**^**)**			
Culture system	2	43.5	< 0.0001
Culture system × light	2	13.7	< 0.0001

Only statistically significant (*p* < 0.01) findings have been included; non-significant results, as well as Tukey’s honestly significant differences and tank effects, can instead be found in Supplementary Table S1. Colony height (cm) was measured in corals of the RAS+B and FTS only (final sampling time only); since these two culture systems were independent, a standard 3-way ANOVA (culture system × light × flow) was instead used. No treatment factor affected base diameter (mm; see Supplementary Table S1 and Supplementary Figs. S1A–D; S2B,C.). Please note that, in Supplementary Figs. S1 and S2, individual one-way ANOVAs for determining the effects of culture system (RAS−B vs. RAS+B vs. FTS) and treatment (the four light × flow interaction groups), respectively, within each treatment and culture system, respectively, were instead carried out. AFDW = ash-free dry weight. ODD = oral disc diameter. SGR = specific growth rate.

^a^square root-transformed data. ^b^log-transformed data. ^c^see Fig. 2D for raw color scores over time for the 12 culture system × treatment groups.

## Discussion

All soft corals in the three culture systems survived, even those cultured for 170 days; this actually represents the longest experimental culture of soft corals (only 30 days–5 months in other studies^14,20^). However, corals grew slowly, and tended to become paler, in the RAS−B system; upon feeding (RAS−B modified to RAS+B), they regained pigmentation, and their ODD enlarged. Furthermore, unlike the ceramic pedestals of the FTS, those of the RAS+B did not regularly become biofouled by algae. This may not only be due to the use of synthetic seawater, in which nutrients were not present, but also because RAS+B coral feeding was undertaken in a separate tank. We therefore avoided the eutrophication associated with feeding experiments carried out in RAS^12^, in which algae may bloom and smother corals; when other studies carried out feeding directly in the RAS^17,26^, such high nutrient loads were generated that soft coral growth was ultimately thwarted. We therefore recommend to those seeking to culture soft corals over long-term timescales to use RAS and feed their organisms in separate tanks.

Fed corals demonstrate lower SGR than starved ones^17^. Herein the SGR of the fed corals in RAS+B was lower than in RAS−B corals for one (HLLF) of the four light × flow interaction groups; however, RAS+B ODD increased more rapidly than in the other two culture systems. The fact that the % increase in ODD (but not AFDW) was higher in RAS+B corals is likely due to heterotrophy^7^. For instance, fed colonies of the symbiotic temperate coral *Astrangia poculata* exhibited significantly greater photosynthetic efficiency than starved conspecifics^29^.

It should be noted that SGR and ODD data gave conflicting results: RAS−B corals grew faster when looking at the former response variable, with ODD change revealing that RAS+B corals grew faster. Given the issues raised in the Introduction with using the BW technique with predominantly soft-bodied organisms, we argue herein that soft coral biologists carefully consider its validity; perhaps the easily measured ODD is a superior, alternative metric for soft coral growth^23,24,26^. Indeed, others have suggested that BW data may be spurious since soft coral tissues contain a significant amount of water, meaning that the density of some colonies may be similar to that of pure seawater^25^. Regardless, the SGR documented herein were comparable to, or even higher than, those reported in other studies of soft corals (Table 3), despite their very small starting sizes. The average annual ODD growth rate was 1.13 cm year^−1^ in the three culture systems, similar to the linear growth rate of 1.0 cm year^−1^ of *Sarcophyton* (0.5–4.9 cm colony diameter) on the Great Barrier Reef^24^. Although our growth rates were higher than those reported in other aquarium studies, it is worth noting that, when comparing husbandry to in situ data, soft corals grew faster on the reef in the lone study that made this comparison^26^; it will be critical, then, to gather field data from our Taiwanese field sites to ensure that aquarium growth rates are comparable to in situ ones.

**Table 3:** Comparison of study species, culture system, specific growth rate (SGR), size, and buoyant weight (BW) of coral fragments in soft coral culture studies.

Species	Culture system	SGR (day^−1^ × 100)	Size	BW (g)	Reference
*Sarcophyton glaucum*	RAS−B	0.069–2.765	11–13 mm	0.243–0.434	Herein
*Sarcophyton glaucum*	RAS+B	0.025–1.072	12–15 mm	0.295–0.526	Herein
*Sarcophyton glaucum*	FTS	0–1.828	13–14 mm	0.367–0.647	Herein
*Sarcophyton cf. glaucum*	RAS+B	0.027–0.028	~ 30 mm		^27^
*Sarcophyton cf. glaucum*	RAS+B	0.035–0.04	~ 40 mm		^20^
*Sarcophyton cf. glaucum*	RAS+B	0.11–0.39^a^	1.5 cm^2^		^17^
*Sarcophyton spp*.	RAS+B	0.055–0.380		0.69–0.782	^14^
*Sarcophyton glaucum*	RAS+B		6 mm	0.0077^c^	^26^
*Sinularia flexibilis*	RAS+B	0.039–0.043	10 cm		^21^
*Sinularia flexibilis*	RAS	0–0.016^b^	5–7 cm	0.014–0.033^a^	^19^
*Sinularia flexibilis*	RAS	0.008–0.019^b^	5–6 cm		^18^

The size data for *Sarcophyton* and *Sinularia flexibilis* have been presented as diameter (or area in cm^2^) and length, respectively.

^a^Values were estimated from the figures. ^b^Average weekly value. ^c^Dry weight.

Unlike RAS−B and FTS corals, RAS+B specimens darkened over the duration of the experiment; this reflects either an increase in Symbiodiniaceae density or pigmentation within their cells (or both) and may indicate that starved endosymbionts of the RAS−B in particular were nitrogen limited (especially given the virtual absence of nitrates and nitrites in the seawater)^5^. However, whether the presumed influx of nitrogen and other nutrients occurred via feeding on the exogenously supplied phytoplankton feed (direct feeding)^4^, feeding on the microbial flora released from the live rocks (indirect feeding), or some other mechanism remains to be determined. It is worth noting that the presence of live rock was associated with higher coral survival rates and Fv/Fm values, as well as fewer bleached specimens in hard coral studies^15^, and we advocate continued research on the role of microbiology in marine animal health and husbandry^1,3,7,8^.

Organic weight (AFDW) is a reliable metric for normalizing soft coral data^25^, particularly when inter-species comparisons are made, and it is thought to better reflect the proportion of fleshy coral tissue. AFDW was only significantly affected by flow rate in the FTS, and percentages documented herein (26–33%) were similar to those reported for congenerics in situ (29%)^24^ and higher than those of cultured conspecifics (20–29%)^17^. On the other hand, *S. glaucum* values showed much larger variation (~ 10 to 55%) in a prior work that also compared different culture conditions and systems^26^.

Light intensity affected the ODD in the RAS−B (100 > 200 μmol quanta m^−2^ s^−1^), and both the ODD and AFDW were affected by flow in the FTS. Water flow reduces the diffusive boundary layer around the coral and influences the supply and uptake of dissolved gasses, nutrients, and food, as well as the removal of sediments and metabolic waste products; collectively, then, higher flow rates should benefit the health and growth of corals^22^, and such has been demonstrated in symbiotic soft corals^24^. Herein, though, corals generally grew faster at the lower of the two flow rates; perhaps exposure to constantly high flow rates of 15 cm s^−1^ results in stress to the polyps or actually sweeps food past too quickly for particles to be effectively captured.

Light more significantly affected growth (as ODD) in corals of the RAS−B. This is likely because these corals were dependent on autotrophy alone for nutrition. In contrast, there were few effects of light intensity or flow velocity in corals of the RAS+B, potentially suggesting that exogenous food supply was sufficient for coral growth; had it not been, the corals may have been more sensitive to changes in their abiotic environment. In the future, it will be worthwhile to determine whether fed soft corals obtain relatively more energy and nutrition from heterotrophy than autotrophy; it can only be stated with the data in hand that fed corals generally outperformed starved ones from assessment of the response variables measured.

## Conclusions

We demonstrated that light and flow effects on soft coral physiology are culture system-dependent. Given that (1) soft corals are mostly fleshly and (2) ODD measurements can be made in only several seconds (versus minutes for buoyant weighing), we advocate assessing both ODD and the BW-based SGR in future studies of soft corals, especially *Sarcophyton*. Soft corals cultured in RAS+B featuring live rocks and an exogenous food supply tended to grow more quickly and presented darker pigmentation. Furthermore, by feeding these corals in a separate tank, macroalgal biofouling was virtually eliminated from the RAS+B husbandry tanks. We therefore recommend the future culture of soft corals in RAS+B systems and advocate that similar such experimental marine animal husbandry approaches be conducted elsewhere (and with additional species), especially given the recently identified need to optimize aquarium husbandry for biobanking/biopreserving marine organisms whose habitats have been compromised by climate change and other anthropogenic stressors^28^.

## Methods

### Animal material

Three *S. glaucum* colonies (ODD =  ~ 30 cm) were collected under Kenting National Park permit 1570001572 (to TYF) at depths of 7–8 m outside the inlet of Taiwan’s third nuclear power plant (21° 57′ 15.7" N, 120° 45′ 21.2" E) in Nanwan Bay, Southern Taiwan. Colonies, which were at least 4–5 m apart, were identified in situ by assessment of their morphological characteristics^6^, quarantined in the husbandry facility of the National Museum of Marine Biology and Aquarium for a week, and acclimated in a 200-L flow-through tank characterized by the following conditions: natural seawater filtered to 5 μm, temperature = 26 ± 1 °C (mean ± standard error for this and all other error terms unless stated otherwise), salinity = 35 ± 1, and PAR = 100 ± 1 μmol quanta m^−2^ s^−1^ (light–dark cycle of 12 h/12 h). After this initial acclimation period, a sterilized scalpel was used to cut fragments (~ 10 mm in diameter and ~ 0.3 g BW) along the edge of the oral disc. Each of the three parent colonies produced 81 fragments, and absorbable polyglycolic acid stitches (VISORB, 1/0)^30^ were used to attach them to 2.7-cm, etched, T-shaped ceramic pedestals (average BW = 5.167 g); these stitches have been shown to be associated with shorter recovery and attachment times than the more commonly used rubber bands^14,20^. The 243 fragments were placed in a 200-L tank under the same conditions as above for recovery and attachment for 2 weeks, and all survived the preparatory processes (Fig. 1A). Of these, 108 were used in the RAS−B, and 72 of these were carried over into the RAS+B. A separate 108 were used in the FTS. All three culture systems are described in detail below. Since 72 of the 216 total fragments were analyzed in a repeated measures fashion (RAS−B modified to RAS+B), a total of 288 measurements were made for the non-destructive response variables described below.

### Culture systems

#### RAS−B

The RAS−B included synthetic seawater: Red Sea salt (Red Sea Aquatics, Ltd.) mixed with reverse osmosis (RO) water (no additional microbial flora). In order to ensure consistent water quality, all six experimental tanks (each 60 × 35 × 20 cm; Fig. 1A) were connected in series to a 240-L “life support” tank (120 × 45 × 45 cm), which contained a 0.2-mm filter bag, an automatic Mato-2009 RO bucket (Autoaqua, Taiwan), a protein skimmer (JNS, CO2, Taiwan), a zeolite drum (JNS, ZR-2), a primary pump (Mr. Aqua, 6000 L/H, Taiwan), a titration system (Johnlen, CS072A-1, Taiwan; for measuring alkalinity [as KH] and concentrations of Ca^2+^ nd Mg^2+^), a heater (ISTA, 350W, USA), and a chiller (Resun, C-1000 p, China; 26 ± 1 °C). The salinity was maintained at 35 using a Mato-100P osmoregulator (Autoaqua) that automatically compensated for evaporative water loss by adding fresh RO water. At the beginning of the experiment, 108 fragments were randomly placed across the 6 experimental tanks (n = 18 fragments/tank), where they were then cultured for 84 days. At the end of this period, 36 fragments were randomly selected and stored in a − 20 °C freezer for analysis of organic matter (AFDW; described below).

#### RAS+B

The remaining 72 fragments were left in the six tanks, and the protein skimmer was removed to keep dissolved and particulate organic material in the synthetic seawater column; it was hypothesized that doing so would enhance the biological activity in the system and potentially benefit the remaining corals^12^. Live rocks (50 kg) were also added because, similarly, it was hypothesized that doing so could increase the growth and color of the soft corals (color paled over the first 84 days in the RAS−B treatment; see Fig. 2B,C). The addition of live rock, an effective biofilter, can improve nutrient cycling and shift microbial communities towards a more typical seawater assemblage^15−16^. Thus, the RAS−B was modified to a RAS+B, and the 60-day RAS+B vs. FTS (see below) experiment began on the 110th day.

In addition to the features listed above, corals of the RAS+B culture system were fed with a reef phytoplankton solution (Seachem, USA) containing concentrated microalgae (*Thalassiosira weissflogii*, *Isochrysis *sp., & *Nannochloropsis *sp.) once every 3 days. After the lights were turned off, fragments were moved into an independent, 20-L feeding tank (60 × 35 × 20 cm) with bubble stones in the four corners (to allow for even water mixing) and a heater that maintained the temperature at 26 °C. After 30 min, the polyps and tentacles extended, and 3 mL of the feeding solution were added. After 4 h, the fragments were removed from the feeding tank, rinsed with filtered seawater, and returned to the experimental tanks. It was hypothesized that, by feeding in a separate tank, the elevation in nutrient levels and macroalgal biomass associated with the feeding process would be avoided.

#### FTS

For these tanks (n = 6), natural seawater was first pumped in from the nearby ocean (Houwan Bay), filtered through sand (50 μm) and passed through 30-ton coral reef mesocosm tanks (described previously^31^) in which seawater temperature was maintained at 26 ± 1 °C. After filtering sequentially (100, 50, 5 μm), the seawater then entered the experimental tanks. The water flow-through (tank volume h^−1^) was 10 L h^−1^, and there was no “life support” tank. The 108 coral fragments were transferred from the acclimation tanks (where they had acclimated for 110 days) to the FTS tanks at the same time as the RAS−B were converted to RAS+B, and the FTS vs. RAS+B comparison was carried out over 60 days (culture days 110–117; see Fig. 2).

### Water quality

Both the RAS−B and RAS+B systems were connected to a 240-L life support tank (315 L in total), and partial synthetic seawater in the RAS−B and RAS+B was changed weekly (30 L; ~ 10% of the volume of the entire culture system). For all three culture systems, concentrations of nutrients (nitrate, nitrite, phosphate, and ammonia), calcium (Ca^2+^), magnesium (Mg^2+^), carbonate hardness/alkalinity (KH), and pH of the three systems were measured weekly (Salifert Profi Test, Holland).

### Light and flow treatments

A PVC plate was placed in the center of each of the six tanks within each of the three culture systems (Fig. 1A), and LED lights (Illumagic, ComboRay G2, Taiwan) were programmed to administer 100 μmol quanta m^−2^ s^−1^ (low light) to one half of each divided tank and 200 μmol quanta m^−2^ s^−1^ to the other (n = 36 experimental areas across the 18 tanks, each at a 12-h/12-h light/dark cycle). Light levels were chosen based on levels used in prior studies that were associated with relatively high soft coral growth rates^17,19−21,26−27^. Each experimental tank also contained a flow motor (Maxspect, GP-03, China), and half of the six tanks were exposed to a flow of 5 cm s^−1^, with the remaining three at a higher flow of 15 cm s^−1^. As for the PAR levels employed, these flow rates were set based on those that were associated with relatively high soft coral growth in prior studies^17,19−21,26−27^.

Thus, there were four treatments in triplicate within each of three culture systems: high light/low flow (HLLF), high light/high flow (HLHF), low light/low flow (LLLF), and low light/high flow (LLHF). The low/high light intensity averaged 105 ± 0.15/206 ± 0.25 μmol quanta m^−2^ s^−1^, and the low/high flow velocity averaged 4.6 ± 0.06/15.7 ± 0.16 cm s^−1^, as measured by light (Li-Cor, LI-193SA, USA) and flow (Kenek, GR20/GR3T-2-20 N, South Korea) meters, respectively. For RAS−B and RAS+B, 9 and 6 soft coral fragments were analyzed in each of the 12 experimental areas (2 areas for each of the 6 experimental tanks), resulting in a total of 108 and 72 fragments for the light × flow experiment, respectively. For the FTS, 9 fragments were used, resulting in a total of 108 fragments.

### BW and SGR

The weights of the coral fragments were measured by a buoyant weighting technique on a Mettler Toledo AB204 balance (precision = 0.1 mg; USA). A glass beaker containing filtered seawater (26 ± 0.5 °C and salinity of 35) and a thermostatic bath were placed under the electronic scale, and the coral fragments were suspended on fishing line under the electronic scale for buoyant weight measurements. Before each measurement, the surface of the coral pedestal was lightly brushed with a toothbrush to remove algae. RAS−B fragment weights were measured every 3 weeks, while RAS+B and FTS fragments were weighed biweekly. The SGR (day^−1^) was calculated as: (lnW_f_ − lnW_i_)/Δt, where lnW_i_ and lnW_f_ represented the natural logarithms of the coral fragment buoyant weights (technically buoyant mass, g, but regularly referred to as “weight” in virtually all publications) at the beginning and the end of the experiment, respectively, and ∆t represented the duration in days. We generally multiplied these data by 100 in most figures. Although we have shown the raw BW data for each culture system (Fig. 2A), we generally prioritized the SGR in our discussion since, unlike raw BW data, it accounts for potential starting size differences across corals.

### ODD

The mean diameters ((length + width)/2) of the oral discs were measured at night (when tentacles had retracted) with vernier calipers (readable to 1 mm) at times = 0 and 84 days for RAS−B. A % change in ODD over this time period was calculated, since, as with BW, a relative rate of change theoretically better accounts for starting size differences across soft corals [as well as the fact that culture durations differed between RAS−B (84 days) and RAS+B and FTS (70 days)]; for this reason, temporal differences in raw ODD across all four treatments have not been depicted for each culture system (data pooled across treatments for each culture system can instead be seen in Fig. 2B, with rates of change found in all following figures). For FTS and RAS+B, measurements were made on culture days 110 and 170, with the % change in ODD also calculated. ODD % change day^−1^ has generally been shortened to “ODD growth” throughout the article, though, for some correlation-based analyses (see below.) raw ODD values were instead used.

### Height and base diameter

The height and mean base diameter ((length + width)/2) of each colony were measured at night when the polyps were retracted with vernier calipers (as above) on culture day 170 (RAS+B and FTS only).

### AFDW

Coral fragments (n = 36, 72, nd 108 for RAS−B, RAS+B, and FTS, respectively) were removed from the pedestals and dried in a vacuum freeze dryer (Xian Toption Lyophilizer, China) for 48 h. The dry weight was measured, after which the samples were incinerated at 450 °C in an MF-30 muffle furnace (Hipoint, Taiwan) for 2 h to calculate the inorganic weight. AFDW was calculated (as a proxy for % organic weight) as (dry weight-inorganic weight)/dry weight × 100.

### Color score

Coral fragments were photographed with a fixed light source (5500 K, LED) in a 40 × 40 × 40 cm studio at the beginning and end of the experiment using a digital camera (Olympus, Tough TG-5). Based on CoralWatch’s “Coral Health Chart”^32^, fragments were scored along the E1 to E6 axis, and the changes in color scores (final assessment level-initial), rather than color itself, were assessed in the statistical tests outlined below to eliminate bias due to certain corals beginning experiments at slightly paler levels than others. That being said, we did plot raw color values over time for each treatment × culture system interaction group (Fig. 2D) to highlight differential effects of treatment on color changes for RAS+B and FTS corals, since, in some cases, starting pigmentation levels were similar.

### Statistical analyses

Data were first tested for normality (Shapiro–Wilk’s test of residuals) and equal variance (Levene’s test), and, since seawater quality parameters did not conform to these assumptions, they were analyzed by non-parametric Kruskal–Wallis tests (on ranks) to determine the effect of culture system on each parameter. Dunn's multiple comparisons post-hoc tests were performed to compare individual culture system means. Because the RAS−B and RAS+B experimental stages were not independent, they were treated as repeated measures (two-way) in the statistical analysis of the physiological response variables for (1) BW (raw values and raw increases per day), (2) SGR (% change day^−1^), (3) % change in ODD day^−1^, (4) organic weight (AFDW; final time only), and (5) color. For these analyses, both the temporal change (excluding AFDW) and the final values were assessed separately. For those two parameters (colony height and base diameter) assessed only in corals of the latter two stages, RAS+B and FTS, the more traditional 3-way ANOVA (culture system × light × flow) was instead carried out since these two culture systems were fully independent. For two-way, repeated measures ANOVAs, tank was nested within light (df = 1) × flow (df = 1), whereas for three-way ANOVA, tank was nested within culture condition (df = 1 since RAS−B data were excluded from these analyses) × light × flow. Tukey’s honestly significant difference (HSD) post-hoc tests were used to compare individual mean differences among culture systems, across treatments, or in response to light and/or flow.

As a less conservative approach, one-way ANOVAs were carried out to test for the effect of culture system for each of the four light × flow treatments (Supplementary Fig. S1) for each of the following response variables: colony height, colony base diameter, SGR, ODD (final values and % changes), organic weight (final values only), and color (final values and raw changes). Similarly, two-way ANOVAs (light × flow) were carried out within each culture system for the same response variables (Supplementary Fig. S2), and one-way repeated measures ANOVAs were used to evaluate the effects of time and culture system on BW, ODD, and color (Fig. 2). Alpha levels of 0.01 and 0.05 were set a priori for ANOVAs and post-hoc tests, respectively.

To depict variation across culture systems within a multivariate framework, a principal components analysis (PCA) on correlations was carried out with standardized data from the following response variables: SGR (% day^−1^), ODD (% change day^−1^), organic weight (%; final values only), and change in color (final-initial values). A canonical correlation analysis (CCA) based on a multivariate ANOVA (MANOVA) was also carried out to uncover multivariate differences between culture systems, and Wilks’ lambda was calculated to assess statistical significance. All statistical analyses were performed using JMP (ver. 14.2).

### Ethics statement

Animals involved in the experiments are not listed in CITES and were cultured in the laboratory for experimental purposes only.

## Supplementary information

Supplementary Information 1.

Supplementary Information 2.
