# Supplementary material for: Culture systems influence the physiological performance of the soft coral Sarcophyton glaucum
Source: Sci Rep. 2020 Nov 19;10:20200. doi: 10.1038/s41598-020-77071-5 (PMC7678846; doi:10.1038/s41598-020-77071-5)
Supplement: Supplementary file 1 — Supplementary Information 1. [file 41598_2020_77071_MOESM1_ESM.docx]

**Supplementary Information**

**Culture systems influence the physiological performance of the soft coral *Sarcophyton glaucum***

Tai-Chi Chang^1^, Anderson B. Mayfield^2,3,4^, & Tung-Yung Fan^1,2*^

^1^Institute of Marine Biology, National Dong Hwa University, Pingtung 944, Taiwan.

^2^National Museum of Marine Biology and Aquarium, Pingtung 944, Taiwan.

^3^Atlantic Oceanographic and Meteorological Laboratory, National Oceanic and Atmospheric Administration, Miami, FL 33149 USA.

^4^Cooperative Institute for Marine and Atmospheric Studies, University of Miami, FL 33149 USA.

Corresponding author: Dr. Tung-Yung Fan (email: tyfan@nmmba.gov.tw)

**Supplementary Results**

**Buoyant weight (BW) & specific growth rate (SGR).** In addition to assessing raw BW increases over time (discussed in the main text & in Fig. 2A), BW was also analyzed as a rate of change ([final-initial]/∆t); this response variable differed across culture systems (Tables 2 & S1). Corals of the RAS-B had a higher total BW increase day^-1^ than conspecifics of the other two groups (data not plotted). In the two-way, repeated measures ANOVA (Tables 2 & S1; with experimental stage as the repeated measure), there were significant effects of culture system, flow x light, & culture system x flow on the SGR; regarding the former, growth was faster (normalized per day) in corals of the RAS-B when data were pooled across all four light x flow treatments. In the high-light+high-flow (HLHF) treatment, the RAS-B mean coral SGR was significantly higher than in soft corals of the FTS (Fig. S1E). When looking across treatments within each culture system (Fig. S2D-F), no significant differences were documented. Because SGR (discussed below) better accommodates differences in starting sizes across experimental fragments, we generally prioritized SGR-based discoveries in the main text.

**Height & base diameter.** Fragment height was significantly affected by flow (Tables 2 & S1) in both the RAS+B (Fig. S1A) and FTS (Fig. S2B): 5>15 cm s^-1^. However, because 1) this parameter was not assessed in corals of the RAS-B and 2) there was a statistically significant tank effect (Table S1), we have not discussed these findings at length (nor were these data included in the PCA & other multivariate analyses). In contrast, neither culture system (Fig. S1A-D) nor treatment (Fig. S2A-C) affected colony base diameter (Tables 2 & S1).

**Oral disc diameter (ODD).** RAS+B coral ODD (as a % change day^-1^) grew significantly faster than conspecifics of the RAS-B and FTS treatments (Tables 2 & S1). This was particularly the case in the high-light treatments, in which RAS-B corals’ ODD actually shrunk on average (Fig. S1I-J). In addition, RAS-B corals’ ODD growth (Fig. S2G) was significantly affected by light (*F*=9.001, *p*=0.004; 100>200 μmol quanta m^-2^ s^-1^ [2-fold]). FTS corals’ ODD growth (Fig. S2I) was instead significantly affected by flow (*F*=11.6, *p*<0.001; 5>15 cm s^-1^ [2-fold]); neither light nor flow affected the ODD growth rate of corals of the RAS+B (Fig. S2H).

**Organic weight.** The ash-free dry weight (AFDW) ranged from 18 to 50% and differed significantly across culture systems (Tables 2 & S1); it was significantly higher in corals of the RAS-B (31.4%±1.0) relative to the other two groups (FTS=28.4%±0.6 & RAS+B=28.2%±0.5). Under low light and high flow conditions (LLHF; Fig. S1O), AFDW was higher in corals of the RAS-B vs. FTS. That said, AFDW was not generally responsive to changes in light or flow for RAS-B (Fig. S2J) or RAS+B (Fig. S2K); only flow significantly affected this parameter in the FTS (Fig. S2L; 5>15 cm s^-1^).

**Color.** The color score of fragments within RAS-B decreased over the 84-day period (Fig. 2C-D & S1Q-T; repeated measures ANOVA effects of time, *p*<0.001), and the pigmentation of corals of the high-light treatments recovered to a lesser extent than those cultured under low light levels (Fig. S2M). In the RAS+B (Fig. 2C-D & S1Q-T), colony pigmentation significantly increased over 60 days for the two high-light treatments (HLHF & HLLF; both *p*<0.001; mean color increase=~0.5). As such, there was a significant effect of culture system on color change (Tables 2 & S1); corals of the RAS+B treatment increased in pigmentation (0.4±0.07), while those of the FTS (-0.1±0.03), and RAS-B (-0.7±0.10) lost pigmentation (Fig. 2C-D).

**Table S1.** Two-way, repeated measures ANOVA results for the effects of culture system (n=3 stages [the repeated measure]), light (100 vs. 200 quanta μmol m^-2^ s^-1^), flow (5 vs. 15 cm s^-1^), and their interaction on soft coral response variables (n=3 per interaction group per culture system). A simpler version of this table featuring only statistically significant (***p*<0.01**; bold font herein) findings has been shown in the main text (Table 2). Colony height (cm) and base diameter (mm) were measured in corals of the RAS+B and FTS only (final sampling time only); since these two culture systems were independent, a standard 3-way ANOVA (culture system x light x flow) was instead used to analyze these parameters (denoted by asterisks “*”). Please note that, in Figs. S1 and S2, individual one-way ANOVAs for determining the effects of culture system (RAS-B vs. RAS+B vs. FTS) and treatment (the four light x flow interaction groups), respectively, within each treatment and culture system, respectively, were instead carried out as a less statistically conservative approach to the composite models presented in this table. AFDW=ash-free dry weight. HSD=honestly significant difference (only select comparisons have been shown for certain interaction effects due to table space constraints). ODD=oral disc diameter. SGR=specific growth rate. In the “Tukey’s HSD (select comparisons only)” column, “200,” “100,” “15,” and “5” following the culture system name correspond to 200 μmol quanta m^-2^ s^-1^, 100 μmol quanta m^-2^ s^-1^, 15 cm s^-1^, and 5 cm s^-1^, respectively.

| **Response variable**  **effect** | | **df** | | **Exact *F*** | ***p*** | **Tukey’s HSD (select comparisons only)**  lowercase letters denote significant differences |
| --- | --- | --- | --- | --- | --- | --- |
| **Buoyant weight (increase day^-1^)^a^ (not plotted across all four light x flow interaction groups)** | | | | | | |
| culture system (see Fig. 2A for raw data.) | | 2 | | 49.1 | **<0.0001** | RAS-B(a)>FTS(b)>RAS+B(c) |
| light | | 1 | | 1.33 | 0.253 |  |
| culture system x light | | 2 | | 6.30 | **0.00330** | RAS-B-200(a)>FTS-100(bc)>RAS+B-100(c) |
| flow | | 1 | | 3.99 | 0.0505 |  |
| culture system x flow | | 2 | | 4.36 | **0.0173** | RAS-B-5(a)>RAS+B-5(b)>RAS+B-15(c) |
| light x flow | | 1 | | 0.0276 | 0.869 |  |
| culture system x light x flow | | 2 | | 1.52 | 0.226 |  |
| tank(light x flow) | | 8 | | 1.00 | 0.444 |  |
| **Colony height* (cm; final value only) (Figs. S1A-D, S2B-C)** | | | | | | |
| culture system (RAS+B vs. FTS only) | | 1 | | 0.617 | 0.434 |  |
| light | | 1 | | 0.726 | 0.395 |  |
| culture system x light | | 1 | | 1.93 | 0.167 |  |
| flow | | 1 | | 63.01 | **<0.0001** | 5(a)>15(b) cm s^-1^ |
| culture system x flow | | 1 | | 7.60 | **0.0065** | FTS-5(a)=RAS+B-5(a)>RAS+B-15(b)=FTS-15(b) |
| light x flow | | 1 | | 0.0515 | 0.821 |  |
| culture system x light x flow | | 1 | | 7.44 | **0.0071** |  |
| tank (culture system x light x flow) | | 16 | | 2.095 | **0.0110** |  |
| **Colony base diameter* (mm; final value only) (Figs. S1A-D, S2B-C)** | | | | | | |
| culture system | 1 | | 3.71 | | 0.0558 |  |
| light | 1 | | 0.0128 | | 0.9101 |  |
| culture system x light | 1 | | 0.0523 | | 0.820 |  |
| flow | 1 | | 1.21 | | 0.273 |  |
| culture system x flow | 1 | | 0.0309 | | 0.861 |  |
| Light x flow | 1 | | 0.567 | | 0.453 |  |
| culture system x light x flow | 1 | | 0.4005 | | 0.528 |  |
| tank(culture system x light x flow) | 16 | | 0.887 | | 0.585 |  |
| **SGR (day^-1^)^b^ (Figs. S1E-H, S2D-F)** | | | | | | |
| culture system | 2 | | 55.6 | | **<0.0001** | RAS-B(a)>RAS+B(ab)=FTS(b) |
| light | 1 | | 1.66 | | 0.2032 |  |
| culture system x light | 2 | | 3.082 | | 0.0535 |  |
| flow | 1 | | 1.24 | | 0.270 |  |
| culture system x flow | 2 | | 5.33 | | **0.0075** | RAS-B-5(a)>RAS+B-15(bc)>RAS+B-5(c) |
| light x flow | 1 | | 10.20 | | **0.0023** | No *post-hoc* differences |
| culture system x light x flow | 2 | | 1.104 | | 0.338 |  |
| tank(light x flow) | 8 | | 0.7068 | | 0.591 |  |
| **ODD (% change day^-1^) (Figs. S1I-L, S2G-I; also, see Fig. 2B for raw data.)** | | | | | | |
| culture system | 2 | | 7.12 | | **0.0017** | RAS+B(a)>FTS(b)=RAS-B(b) |
| light | 1 | | 0.263 | | 0.610 |  |
| culture system x light | 2 | | 3.61 | | **0.0334** |  |
| flow | 1 | | 0.370 | | 0.546 |  |
| culture system x flow | 2 | | 0.341 | | 0.713 |  |
| light x flow | 1 | | 0.0827 | | 0.775 |  |
| culture system x light x flow | 2 | | 0.0559 | | 0.946 |  |
| tank(light x flow) | 8 | | 0.917 | | 0.5095 |  |
| **Percent organic weight (AFDW)^c^ (Figs. S1M-P, S2J-L)** | | | | | | |
| culture system | 2 | | 14.2 | | **<0.0001** | RAS-B(a)>FTS(b)=RAS+B(b) |
| light | 1 | | 0.0944 | | 0.761 |  |
| culture system x light | 2 | | 0.145 | | 0.866 |  |
| flow | 1 | | 1.33 | | 0.261 |  |
| culture system x flow | 2 | | 1.55 | | 0.234 |  |
| light x flow | 1 | | 0.0361 | | 0.851 |  |
| culture system x light x flow | 2 | | 1.29 | | 0.296 |  |
| tank(light x flow) | 8 | | 1.23 | | 0.328 |  |
| **Color change (final-initial) (Figs. S1Q-T, S2M-O^c^)** | | | | | | |
| culture system (Figure 2C) | 2 | | 43.5 | | **<0.0001** | RAS+B(a)>FTS(b)>RAS-B(c) |
| light | 1 | | 2.50 | | 0.120 |  |
| culture system x light | 2 | | 13.7 | | **<0.0001** | RAS+B-200 μmol quanta m^-2^ s^-1^> all others |
| flow | 1 | | 0.0581 | | 0.810 |  |
| culture system x flow | 2 | | 1.79 | | 0.176 |  |
| light x flow | 1 | | 0.0868 | | 0.769 |  |
| culture system x light x flow | 2 | | 0.527 | | 0.593 |  |
| tank(light x flow) | 8 | | 0.475 | | 0.869 |  |

^a^square root-transformed data. ^b^log-transformed data. ^c^log-transformed data. ^c^see Fig. 2D for raw color scores over time for the 12 culture system x treatment groups.

**Supplemental figures**


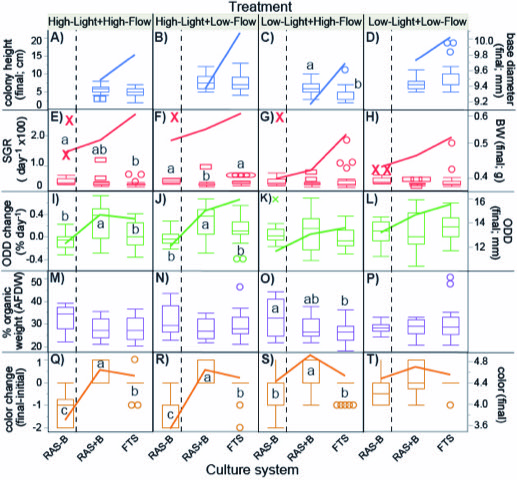


**Fig. S1.** Physiological responses of soft corals cultured in three different systems- RAS-B, RAS+B, and FTS- exposed to one of four experimental treatments: high light+high flow (HLHF; A,E, I, M, & Q), high light+low flow (HLLF; B, F, J, N, & R), low light+high flow (LLHF; C, G, K, O, & S), and low light+low flow (LLLF; D, H, L, P, & T). With the exception of height (box plots of A-D; left y-axis), colony base diameter (connected, solid lines of A-D; right y-axis), and percent (%) organic weight (i.e., ash-free dry weight) data (box plots of M-P), which represent final sampling time values only, the left and right y-axes represent the changes (box plots) and final values (connected, solid lines), respectively, of the following response variables: specific growth rate (SGR; E-H), oral disc diameter (ODD; I-L), and fragment color (Q-T). Lowercase letters above/adjacent to box plots represent Tukey’s honestly significant differences (*p*<0.05) between culture systems for the net change (i.e., left y-axis) data only. Outlier data points for RAS-B, RAS+B, and FTS are denoted by exes, squares, and circles, respectively, and a hatched, vertical line has been placed between the RAS-B and RAS+B data to emphasize the fact that these samples were not statistically independent.


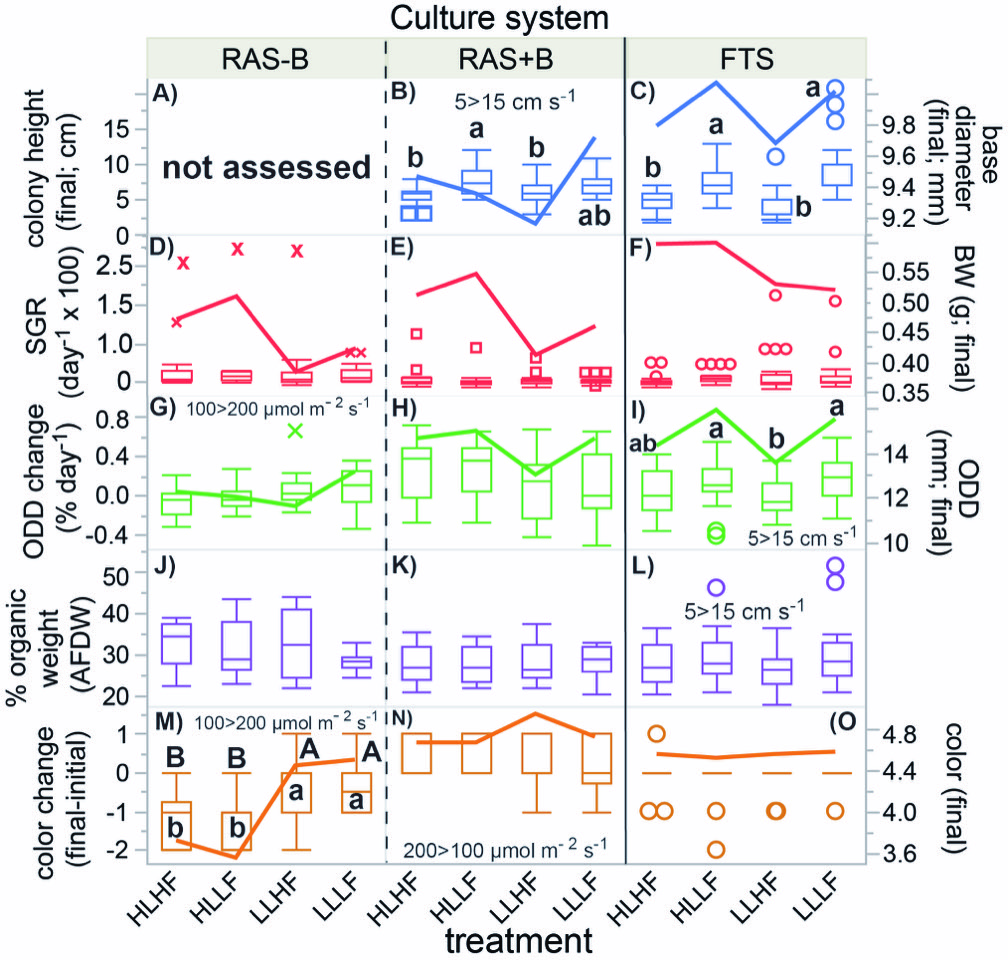


**Fig. S2.** Physiological responses of soft corals cultured in three different systems- RAS-B (A, D, G, J, & M), RAS+B (B, E, H, K, & N), and FTS (C, F, I, L, & O [the latter being offset to accommodate certain data points])- exposed to one of four experimental treatments: high-light+high flow (HLHF), high-light+low flow (HLLF), low-light+high flow (LLHF), and low-light+low flow (LLLF). With the exception of the colony height (box plots of A-C; left y-axis), colony base diameter (connected, solid lines of A-C; right y-axis), and percent (%) organic weight (i.e., ash-free dry weight) data (box plots of J-L), which represent final sampling time values only, the left and right y-axes represent the changes (box plot) and final values (connected, solid lines), respectively, of the following response variables: specific growth rate (SGR; D-F), oral disc diameter (ODD; G-I), and fragment color (M-O). Lowercase letters adjacent to box plots represent Tukey’s honestly significant differences (*p*<0.05) between experimental treatments for the net change (i.e., left y-axis) data only, whereas capital letters connotate right y-axis (i.e., final measurement time) differences. Significant differences (*p*<0.01) across light and/or flow that were detected by two-way ANOVA and discussed in the main text have been included in certain panels. Outlier data points for RAS-B, RAS+B, and FTS are denoted by exes, squares, and circles, respectively, and a hatched, vertical line has been placed between the RAS-B and RAS+B data to emphasize the fact that these samples were not statistically independent.
